# Characteristics, Complications, Comorbidities, and Other Manifestations of Inflammatory Bowel Disease: A 7-Year Tertiary Center Experience

**DOI:** 10.3390/clinpract16030045

**Published:** 2026-02-24

**Authors:** Waleed Alharbi, Turki Alasmari, Najla Al Rasheed, Jamila A. Alonazi, Naif K. Alaqil, Meshari Al Samih, Nawaf S. Alzahrani, Abdulaziz Bin Akrish, Soliman Alaraidh

**Affiliations:** 1 College of Medicine, King Saud bin Abdulaziz University for Health Sciences, Riyadh 11481, Saudi Arabia; 2 King Abdullah International Medical Research Center, Riyadh 11481, Saudi Arabia; 3 Department of Internal Medicine, King Abdulaziz Medical City Riyadh, Riyadh 11426, Saudi Arabia; 4 Department of Family Medicine, King Abdulaziz Medical City Riyadh, Riyadh 11426, Saudi Arabia

**Keywords:** IBD, Crohn’s disease, ulcerative colitis, multimorbidity, hospitalization

## Abstract

**Background/Objectives**: Inflammatory bowel disease (IBD) is associated with significant morbidity worldwide. While global epidemiological trends are well-documented, data on the clinical and demographic characteristics of IBD patients in Saudi Arabia remain limited. This study aimed to evaluate the distribution of multimorbidity among IBD patients in a tertiary Saudi hospital and assess associated clinical features and outcomes. **Methods**: A retrospective cross-sectional study of IBD patients treated at the National Guard Hospital over a seven-year period was conducted. Data on demographics, body mass indices (BMIs), hospitalizations, comorbidities, complications, and surgical interventions were extracted from medical records. Associations between categorical and continuous variables were analyzed using chi-square and t-tests, respectively, with significance being set to *p* < 0.05. **Results**: A total of 465 patients were included: 54.6% had Crohn’s disease (CD) and 45.4% had ulcerative colitis (UC). CD predominated in males (60.6%), while UC was more common in females (55.5%, *p* = 0.001). BMI distribution differed significantly between groups (*p* = 0.004). Hospital admission rates and length of stay were higher among CD patients (*p* = 0.032). CD patients experienced greater complication rates, including fistulas (41.3% vs. 7.1%, *p* < 0.001) and strictures (26.1% vs. 1.4%, *p* < 0.001). Surgical interventions such as fistulotomy (4.3% vs. 0.5%, *p* = 0.009) and stricturoplasty (9.1% vs. 1.9%, *p* = 0.001) were more frequent in patients with CD. **Conclusions**: This study characterizes IBD patients in Saudi Arabia, highlighting gender differences, BMI variations, and the greater severity of CD compared with UC. The higher rates of complications and surgical interventions among CD patients emphasize the need for tailored management strategies. Future prospective studies are warranted to investigate disease progression and optimize care for this population.

## 1. Introduction

Multimorbidity is defined as co-occurrence of at least two chronic conditions in the same individual [1]. Multimorbidity is a significant global issue that is constantly growing and impacting patients, caregivers, society, and health systems and resource poor settings in particular [2,3]. Multimorbidity is distinct from the similar concept of comorbidity, which refers to the combined effects of additional conditions in relation to the primary condition in an individual [4,5]. In contrast, multimorbidity care is patient-centered and does not habitually prioritize any particular ailment, while in clinical care, patients and doctors will normally focus on the patient’s most pressing difficulties.

In contrast to people with a single chronic condition, people with multimorbidity have a higher probability of dying prematurely, experience constant hospital admissions and have an increased length of stay [6,7]. Multimorbidity is also associated with poorer function and health-related quality of life (HRQoL), depression, intake of multiple drugs (polypharmacy), and greater socioeconomic costs [8,9,10]

Inflammatory bowel disease (IBD) is signalized by repeated episodes of inflammation of the gastrointestinal tract caused by an abnormal immune response to gut microflora [11]. IBD encompasses two idiopathic conditions that affect the intestines; they are differentiated by their depth, involvement of the bowel wall, character, and location of inflammation [12].

Ulcerative colitis (UC) involves disseminated inflammation of the colonic mucosa. Almost always, UC affects the rectum in the form of proctitis, but it can manifest in the sigmoid (proctosigmoiditis), beyond the sigmoid (distal ulcerative colitis), or include the entire colon up to the cecum (pancolitis) [13]. Crohn’s disease (CD) results in transmural ulceration of any part of the gastrointestinal tract (GI), most often affecting the terminal ileum and colon. Both diseases are classified by extent (mild, moderate, or severe) and location. CD is also classified by inflammatory phenotype, structuring, and penetration [14].

In the mid-1990s, Hudson and 6 colleagues were among the first to consider IBD as an independent factor for increased cardiovascular risk [15]. Along with an increased prevalence of nonalcoholic fatty liver disease (NAFLD), obesity, chronic fatigue, and erectile dysfunction have been shown in patients with IBD [16,17,18]. Another paper showcased how common comorbidities among IBD patients are, as 78% of IBD patients had at least one comorbidity, with a median of three comorbidities [19]. As this showcases the global burden on healthcare systems around the world, especially in Saudi Arabia, or even the greater Middle East, we lack literature that targets and showcases multimorbidity in this region.

It warrants further effort and investigation into the subject; therefore, we aim to study the prevalence of multimorbidity in IBD patients and illustrate the association between multimorbidity and IBD patient characteristics and care outcomes in a tertiary care center in Riyadh, Kingdom of Saudi Arabia.

## 2. Materials and Methods

A retrospective cross-sectional study was conducted at National Guard Health Affairs (NGHA) Hospitals in Saudi Arabia. NGHA is a well-established healthcare institution offering a broad spectrum of clinical, academic, and research services, ranging from public health and primary care to specialized tertiary care. This study aimed to analyze the clinical characteristics and disease patterns among patients diagnosed with inflammatory bowel disease (IBD). Data were collected through a systematic chart review using a structured data collection sheet. The study population included Saudi adolescents and adults diagnosed with IBD between January 2016 and September 2022. Patients aged between 15 and 70 years were included in the study, while those aged 14 years and younger were excluded.

The study included all adult patients (≥18 years) with a confirmed diagnosis of Crohn’s disease or ulcerative colitis established through clinical evaluation, endoscopic findings, and radiologic or histopathologic confirmation. Only patients who visited gastroenterology, internal medicine, and general surgery clinics between January 2016 and December 2024 and had complete medical records were eligible. Individuals with active or follow-up visits were included, provided their clinical, laboratory, and imaging data were available for analysis. Patients were excluded if they had an indeterminate or unconfirmed diagnosis of inflammatory bowel disease. Patients younger than 18 years or those who received care primarily at another institution with insufficient documentation were not included in the analysis.

The study utilized a non-probability consecutive sampling technique, as all eligible patients meeting the inclusion and exclusion criteria were included due to the limited number of subjects. Data were extracted from the Best Care system, an electronic medical records platform used at NGHA, to ensure the accuracy and completeness of the variables. The variables collected included demographic characteristics such as age, gender, and body mass index (BMI), as well as clinical variables such as type of IBD based on the Montreal IBD classification, history of thromboembolism, disease relapse and severity, family history of IBD, smoking status, extraintestinal manifestations, hospital readmissions, age at disease onset, inflammatory complications, presence of primary sclerosing cholangitis, and biologic medication usage. The data were collected from Best Care system over the period between 19 April 2024 and 3 August 2024. Comorbidities were identified and extracted using International Classification of Diseases (ICD-10) diagnostic codes recorded at the time of each patient’s clinic visit or hospitalization. For each patient, all active ICD-10 codes listed in problem lists and encounter diagnoses were reviewed.

## 3. Results

A total of 465 patients were included in the study, with 54.6% being diagnosed with Crohn’s disease (CD) and 45.4% with ulcerative colitis (UC). The mean age of the total sample was 38.40 years old with a standard deviation (SD) of 14.60 years. The gender distribution showed a slightly higher proportion of males (53.3%) than females (46.7%). The majority of patients were Saudi nationals (97.2%), while only 2.8% were non-Saudis. Regarding medication usage, the distributions of non-biological and biological treatments were nearly equal, with 49.6% receiving non-biological therapy and 50.4% receiving biological therapy. Hospital admission duration varied among patients, with 47.7% never being admitted, 8.8% being admitted for 1–3 days, 19.7% for 4–7 days, 13.5% for 8–14 days, 7.1% for 15 days to one month, and 3.4% for more than one month (Table 1).

**Figure 1:**
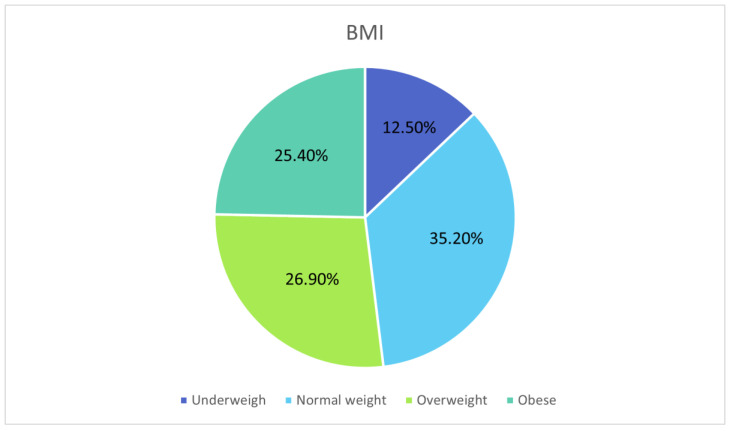
Distribution of patients according to their BMI. Percentages may not sum to exactly 100% due to rounding to one decimal place.

Among the total cohort, 12.5% were classified as underweight, 35.3% as having a normal weight, 26.9% as overweight, and 25.4% as obese. This distribution highlights the significant variation in BMI among patients with inflammatory bowel disease (IBD) (Figure 1).

The data collected were managed and analyzed using Statistical Package for Social Sciences (SPSS) version 25. Prior to analysis, data cleaning and coding were performed to ensure consistency and accuracy. Descriptive statistics were used to summarize categorical variables as frequencies and percentages, while continuous variables were expressed as means with standard deviations. To compare means and proportions, Student’s t-test and chi-square (χ^2^) tests were employed, respectively. Additionally, multivariate logistic regression analysis was conducted to identify factors associated with disease outcomes, including variables that demonstrated statistical significance at the bivariate level. A *p*-value of less than 0.05 was considered statistically significant for all analyses.

Higher comorbidity burden was significantly associated with older age (*p* < 0.001). Female patients were more likely to have ≥3 comorbidities compared with males (*p* = 0.002), and the prevalence of obesity increased progressively with comorbidity burden (*p* = 0.006). Use of non-biological therapy was more frequent among patients with a higher number of comorbidities (*p* = 0.003). Patients with ≥3 comorbidities experienced significantly longer hospital stays (*p* = 0.017), and readmission rates differed significantly across comorbidity groups (*p* = 0.019). Nationality was not associated with comorbidity burden. Crohn’s disease was more common among patients with a single comorbidity, whereas ulcerative colitis predominated in those with ≥3 comorbidities (*p* = 0.029) (Table 2).

Anatomical involvement differed significantly by IBD type, with Crohn’s disease predominantly affecting the small intestine or presenting with combined intestinal involvement, whereas ulcerative colitis was largely confined to the large intestine (*p* < 0.001). Extra-intestinal anatomical involvement was more frequently observed in ulcerative colitis, particularly rectal involvement (*p* < 0.001). Metabolic comorbidities, including diabetes mellitus, hyperlipidemia, and hypertension, were significantly more prevalent among patients with ulcerative colitis compared with those with Crohn’s disease (*p* = 0.011, *p* < 0.001, and *p* = 0.004, respectively). Endocrine disorders, most notably hypothyroidism, were also more common in ulcerative colitis (*p* = 0.002). In addition, primary sclerosing cholangitis demonstrated a strong association with ulcerative colitis (*p* < 0.001). Smoking was significantly more prevalent among patients with Crohn’s disease (*p* = 0.007), whereas stroke occurred more frequently in patients with ulcerative colitis (*p* = 0.014) (Table 3).

**Figure 2:**
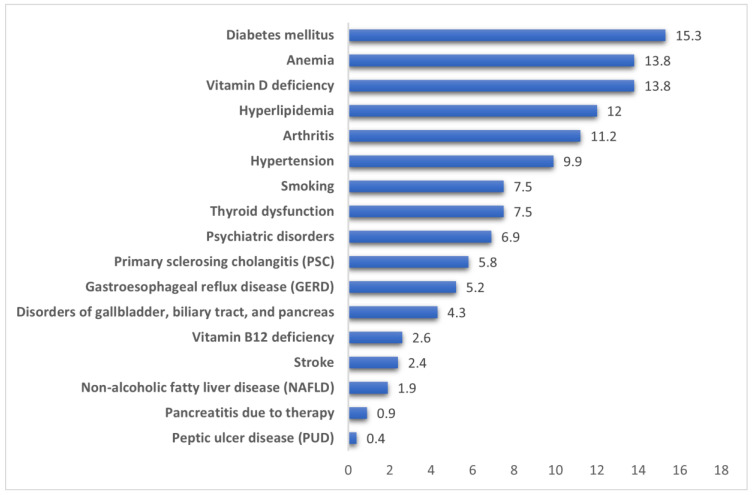
Frequency of comorbidities.

The most common comorbidity among the patients was diabetes mellitus affecting 15.3% of the cohort. Other frequently reported conditions included anemia (13.80%), vitamin D deficiency (13.8%), and hyperlipidemia (12.0%). Hypertension was present in 9.9% of cases, followed by arthritis (11.2%) and psychiatric disorders (6.9%). Smoking was reported in 7.5% of patients. Autoimmune disorders such as psoriasis, celiac disease, and systemic lupus erythematosus (SLE) were uncommon, with respective prevalences of 2.8%, 1.7%, and 1.1% (Figure 2).

CD patients had a significantly higher prevalence of perianal fistulas (18.1%) compared to UC patients (4.3%) (*p* < 0.001). Similarly, hemorrhoids were more common among CD patients (16.1%) than UC patients (9.0%) (*p* = 0.022). Anal abscesses were also significantly more frequent in CD patients (4.7%) compared to UC patients (1.4%) (*p* = 0.045). However, there were no significant differences between the two groups regarding anal fissures and anal strictures (Table 4).

CD patients exhibited a significantly higher incidence of complications compared to UC patients. Fistulas were more prevalent among CD patients (41.3%) compared to UC patients (7.1%) (*p* < 0.001). Similarly, strictures were significantly more common in CD patients (26.1%) than in UC patients (1.4%) (*p* < 0.001). Abscesses and bowel obstruction were also more frequent in CD patients, with abscesses occurring in 10.6% of CD cases compared to 1.4% in UC (*p* < 0.001) and bowel obstruction affecting 6.3% of CD patients versus 0.5% of UC patients (*p* = 0.001). Perforations were observed in 3.9% of CD cases compared to 0.9% of UC cases (*p* = 0.043). No significant differences were noted for adhesions and polyps between the two groups (Table 5).

CD patients had significantly higher rates of fistulotomy (4.3% vs. 0.5%, *p* = 0.009) and stricturoplasty (9.1% vs. 1.9%, *p* = 0.001) compared to UC patients. Hemicolectomy was performed more frequently in UC patients (4.3%) than in CD patients (0.8%) (*p* = 0.014). Proctocolectomy was exclusively performed in CD patients (2.4%) but not in UC patients (*p* = 0.025). No significant differences were found in ileocecal resection, total colectomy, subtotal colectomy, small bowel resection, or abscess drainage between the two groups (Table 6).

The associations between comorbidities (diabetes, hyperlipidemia, hypertension, and pancreatitis due to therapy) and the type and duration of hospital admission were analyzed. The duration of the longest hospital admission varied based on the presence of comorbidities. Notably, patients without diabetes had a higher percentage of not being admitted (55.1%), whereas those with type 1 diabetes had a slightly higher percentage (58.6%). However, all patients with type 2 diabetes required hospitalization for at least four days. The differences in admission duration among diabetes groups were not statistically significant (*p* = 0.316). Similarly, no significant associations were found between admission duration and hyperlipidemia (*p* = 0.146), hypertension (*p* = 0.352), or pancreatitis due to therapy (*p* = 0.066).

Regarding surgical interventions, hemicolectomy and subtotal colectomy showed significant associations with diabetes (*p* < 0.001), while ileocecectomy was significantly associated with pancreatitis due to therapy (*p* = 0.001). Other surgical procedures, such as fistulotomy, stricturoplasty, abscess drainage, ileocecal resection, and small bowel resection, did not show significant associations with any of the analyzed comorbidities (Table 7).

## 4. Discussion

The present study provided a comprehensive analysis of the clinical and demographic characteristics of inflammatory bowel disease (IBD) patients over the past three decades at a single referral center in Saudi Arabia. Our findings highlighted important differences in disease patterns, comorbidities, and treatment modalities between Crohn’s disease (CD) and ulcerative colitis (UC) patients, reflecting broader trends observed in global epidemiological studies.

The predominance of CD (54.6%) over UC (45.4%) in our cohort aligned with some regional studies suggesting an increasing prevalence of CD in Middle Eastern populations [19,20]. This distribution contrasted with Western populations, where UC had traditionally been more prevalent than CD [21]. The gender distribution revealed a male predominance (53.3%), with significant differences between CD and UC patients. CD was more common in males (60.6%), whereas UC was more prevalent in females (55.5%). This gender-based variation was consistent with prior research indicating a higher prevalence of CD in men and UC in women in some populations, possibly due to hormonal, genetic, and environmental factors [22,23].

As per our findings, patients with ≥3 comorbidities had longer hospital stays (*p* = 0.017). However, readmission rates differed significantly across comorbidity groups (*p* = 0.019). As reported by prior literature, predictive factors of readmission for ulcerative colitis were younger age, male sex, and transfusion [24]. For Crohn’s disease, chronic pain and younger age were predictive factors for readmission [25,26].

Obesity increased with comorbidity burden (*p* = 0.006). Moreover, according to prior literature, obesity is a factor associated with increased early readmission with higher burden, with prevention of obesity being a cornerstone to decrease readmission and health care burden [27]. In contrast, obesity was equally distributed among both CD and UC patients, which is consistent with emerging evidence suggesting a rising prevalence of obesity in IBD populations due to lifestyle changes and corticosteroid use [28].

Hospital admission patterns varied significantly between CD and UC patients. A higher proportion of UC patients had never been hospitalized (63.0%) compared to CD patients (49.2%), whereas CD patients experienced more prolonged hospital stays, with 4.7% requiring admission for over a month. This aligned with studies indicating that CD is associated with more severe disease progression, requiring frequent hospitalizations and surgical interventions [29,30].

Regarding anatomical involvement, a substantial proportion of CD patients exhibited both small and large intestine involvement (52.8%), reflecting the characteristic nature of CD as a transmural disease. UC is confined to patient’s large intestine. Extra-intestinal involvement was relatively uncommon, with rectal involvement being the most frequently reported (17.0%), followed by stomach (3.4%) and esophageal involvement (1.3%). These findings were consistent with previous literature, which emphasizes the heterogeneity of IBD manifestations and the potential for extra-intestinal complications [29].

Comorbid conditions were prevalent, with anemia (26.5%) being the most common, followed by endocrine disorders (22.6%) and vitamin D deficiency (13.8%). Anemia is a well-documented complication of IBD that is often attributed to chronic inflammation, gastrointestinal bleeding, and micronutrient deficiencies [31]. Endocrine disorders, particularly vitamin D deficiency, have been frequently reported in IBD patients and are thought to be linked to disease activity and immune dysregulation [32]. Adding on that, IBD and Type 2 Diabetes Mellitus have been linked throughout many studies. Furthermore, some studies described the association to be linked with steroid use while others determined that there was a correlation regardless of steroid use [33,34], which shows that more light needs to be shed on the matter.

A significant finding in our study was the higher prevalence of perianal fistulas (18.1% vs. 4.3%, *p* < 0.001), strictures (26.1% vs. 1.4%, *p* < 0.001), and absences (10.6% vs. 1.4%, *p* < 0.001) in CD patients compared to UC patients. This is in accordance with prior studies highlighting the aggressive nature of CD, which often necessitates surgical interventions due to complications such as fistulas, strictures, and abscess formation [35]. Additionally, CD patients underwent significantly higher rates of stricturoplasty (9.1% vs. 1.9%, *p* = 0.001) and fistulotomy (4.3% vs. 0.5%, *p* = 0.009), reinforcing the need for surgical management in refractory cases [36].

These findings emphasize the need for a multidisciplinary approach in managing IBD patients with comorbidities, as metabolic disorders can influence disease outcomes and surgical risks.

This study has several strengths, including a large sample size and a long-term retrospective analysis. However, limitations such as single-center design and retrospective nature may impact generalizability. Future multicenter studies are needed to validate our findings and explore additional factors influencing IBD progression in the Saudi population.

## 5. Conclusions

In conclusion, our findings provide valuable insights into the clinical, comorbidity, and disease burden characteristics of IBD patients in Saudi Arabia. The higher prevalence of CD, its association with more severe complications, and the significant role of comorbidities highlight the need for comprehensive disease management strategies. Further research should focus on identifying risk factors contributing to disease severity and optimizing therapeutic approaches to improve patient outcomes.

Limitations:

While consecutive sampling and EMR extraction provide a practical and real-world overview of IBD patient characteristics, the findings should be interpreted in light of potential selection bias, missing data, and documentation variability inherent to retrospective designs.

## Figures and Tables

**Table 1:** Baseline characteristics of the study population (N = 465).

		Frequency N (%)
Gender	Female	217 (46.7)
	Male	248 (53.3)
Age (Years)	Mean (SD)	38.4 (14.6)
BMI	Underweight	58 (12.5)
	Normal weight	164 (35.3)
	Overweight	125 (26.9)
	Obese	118 (25.4)
Nationality	Saudi	452 (97.2)
	Non-Saudi	13 (2.8)
Medication Type	Non-biological	230 (49.6)
	Biological	234 (50.4)
Duration of Longest Admission	Not admitted	222 (47.7)
	1–3 days	41 (8.8)
	4–7 days	90 (19.4)
	8–14 days	63 (13.5)
	15 days–1 month	33 (7.1)
	>1 month	16 (3.4)
Diagnosis	Crohn’s disease (CD)	254 (54.6)
	Ulcerative colitis (UC)	211 (45.4)

**Table 2:** Demographic and clinical characteristics of IBD patients stratified by comorbidity burden.

		Comorbidities in IBD Patients			Sig. Value
		Patients with 1 Comorbidity N (%)	Patients with 2 Comorbidities N (%)	Patients with ≥3 Comorbidities N (%)	
Gender	Female	51 (41.5)	32 (39.0)	77 (60.6)	0.002
	Male	72 (58.5)	50 (61.0)	50 (39.4)	
Age (Years)	Mean (SD)	32.6 (10.7)	38.8 (13.3)	49.3 (16.4)	<0.001
BMI	Underweight	21 (17.1)	11 (13.4)	9 (7.1)	0.006
	Normal weight	45 (36.6)	29 (35.4)	38 (29.9)	
	Overweight	32 (26.0)	26 (31.7)	30 (23.6)	
	Obese	25 (20.3)	16 (19.5)	50 (39.4)	
Nationality	Saudi	117 (95.1)	81 (98.8)	125 (98.4)	0.173
	Non-Saudi	6 (4.9)	1 (1.2)	2 (1.6)	
Medication Type	Non-biological	51 (41.5)	43 (52.4)	80 (63.0)	0.003
	Biological	72 (58.5)	39 (47.6)	47 (37.0)	
Longest Admission Duration	Not admitted	56 (45.5)	45 (54.9)	70 (55.1)	0.017
	1–3 days	8 (6.5)	13 (15.9)	7 (5.5)	
	4–7 days	32 (26.0)	8 (9.8)	17 (13.4)	
	8–14 days	15 (12.2)	10 (12.2)	14 (11.0)	
	15 days–1 month	9 (7.3)	3 (3.7)	13 (10.2)	
	>1 month	3 (2.4)	3 (3.7)	6 (4.7)	
Readmission	No	80 (65.0)	67 (81.7)	97 (76.4)	0.019
	Yes	43 (35.0)	15 (18.3)	30 (23.6)	
Condition	CD	72 (58.5)	40 (48.8)	53 (41.7)	0.029
	UC	51 (41.5)	42 (51.2)	74 (58.3)	

**Table 3:** Association of clinical characteristics and comorbidities with IBD types among patients.

		Type of IBD			Sig. Value
		CD N (%)	UC N (%)	Total N (%)	
Anatomical involvement	No data	7 (2.8)	15 (7.1)	23 (4.9)	<0.001
	Small intestine	55 (21.7)	12 (5.7)	67 (14.4)	
	Large intestine	22 (8.7)	139 (65.9)	161 (34.6)	
	Both	134 (52.8)	14 (6.6)	148 (31.8)	
	Unspecified	36 (14.2)	30 (14.2)	66 (14.2)	
Extra-intestinal anatomical involvement	None	212 (83.5)	142 (67.3)	354 (76.1)	<0.001
	Esophagus	4 (1.6)	2 (0.9)	6 (1.3)	
	Stomach	8 (3.1)	8 (3.8)	16 (3.4)	
	Rectum	22 (8.7)	57 (27.0)	79 (17.0)	
	Oral	8 (3.1)	2 (0.9)	10 (2.2)	
Extra-GI manifestations	None	228 (89.8)	184 (87.2)	412 (89.2)	0.110
	Hepatobiliary	3 (1.2)	12 (5.7)	15 (3.3)	
	Ocular	2 (0.8)	2 (0.9)	4 (0.9)	
	Skin	4 (1.6)	1 (0.5)	5 (1.1)	
	MSK	11 (4.3)	7 (3.3)	18 (3.1)	
	Others	7 (2.75)	3 (1.4)	10 (2.2)	
GI Infection	None	227 (89.4)	181 (85.8)	408 (87.7)	0.483
	*H. pylori*	6 (2.4)	3 (1.4)	9 (1.94)	
	*C. difficile*	15 (5.9)	16 (7.6)	31 (6.7)	
	Tuberculosis	3 (1.2)	2 (0.9)	5 (1.1)	
	CMV	2 (0.8)	6 (2.8)	8 (1.7)	
	Salmonella	1 (0.4)	1 (0.5)	2 (0.4)	
	*C. perfringens*	0 (0.0)	1 (0.5)	1 (0.2)	
	Brucellosis	0 (0.0)	1 (0.5)	1 (0.2)	
Renal disease	None	248 (97.6)	199 (94.3)	447 (96.1)	0.142
	CKD	6 (2.4)	11 (5.2)	17 (3.7)	
	Nephrotic	0 (0.0)	1 (0.5)	1 (0.2)	
Arthritis	No	229 (90.2)	184 (87.2)	413 (88.8)	0.314
	Yes	25 (9.8)	27 (12.8)	52 (11.2)	
Diabetes	No	225 (88.6)	169 (80.1)	394 (84.7)	0.011
	Yes	29 (11.4)	42 (19.9)	71 (15.3)	
Endocrine disorders	None	246 (96.9)	186 (88.2)	432 (93.1)	0.002
	Hypothyroidism	6 (2.4)	20 (9.5)	26 (5.6)	
	Hyperthyroidism	2 (0.8)	4 (1.9)	6 (1.3)	
Hyperlipidemia	No	240 (94.5)	169 (80.1)	409 (88.0)	<0.001
	Yes	14 (5.5)	42 (19.9)	56 (12.0)	
Hypertension	No	238 (93.7)	181 (85.8)	419 (90.1)	0.004
	Yes	16 (6.3)	30 (14.2)	46 (9.9)	
Anemia	No	193 (76.0)	149 (70.6)	342 (73.55)	0.191
	Yes	61 (24.0)	62 (29.4)	123 (26.45)	
Vitamin D deficiency	No	219 (86.2)	182 (86.3)	401 (86.24)	0.991
	Yes	35 (13.8)	29 (13.7)	64 (13.76)	
Vitamin B12 deficiency	No	248 (97.6)	205 (97.2)	453 (97.42)	0.744
	Yes	6 (2.4)	6 (2.8)	12 (2.58)	
Primary sclerosing cholangitis	No	251 (98.8)	187 (88.6)	438 (94.19)	<0.001
	Yes	3 (1.2)	24 (11.4)	27 (5.81)	
Smoking	No	222 (87.4)	199 (94.3)	425 (91.4)	0.007
	Yes	26 (10.2)	8 (3.8)	40 (8.6)	
Autoimmune diseases	No	248 (97.6)	204 (96.7)	452 (97.20)	0.507
	Psoriasis	4 (1.6)	4 (1.9)	8 (1.72)	
	Celiac disease	1 (0.4)	3 (1.4)	4 (0.86)	
	SLE	1 (0.4)	0 (0.0)	1 (0.22)	
Stroke	No	252 (99.2)	202 (95.7)	454 (97.63)	0.014
	Yes	2 (0.8)	9 (4.3)	11 (2.37)	
Psychiatric disorders	No	237 (93.3)	196 (92.9)	433 (93.12)	0.860
	Yes	17 (6.7)	15 (7.1)	32 (6.88)	

**Table 4:** Differences between CD and UC patients considering Hemorrhoid and perianal conditions.

		Diagnosis				
		CD		UC		*p*-Value
		Count	Column N %	Count	Column N %	
Perianal Fistula	No	208	81.9%	202	95.7%	<0.001
	Yes	46	18.1%	9	4.3%	
Hemorrhoid	No	213	83.9%	192	91.0%	0.022
	Yes	41	16.1%	19	9.0%	
Anal Fissure	No	249	98.0%	208	98.6%	0.652
	Yes	5	2.0%	3	1.4%	
Anal Abscess	No	242	95.3%	208	98.6%	0.045
	Yes	12	4.7%	3	1.4%	
Anal stricture	No	252	99.2%	210	99.5%	0.674
	Yes	2	0.8%	1	0.5%	

**Table 5:** Association of perianal conditions, complications, and surgical procedures with IBD subtype (Crohn’s disease vs. ulcerative colitis).

		IBD Types		Sig. Values
		CD N (%)	UC N (%)	
Hemorrhoid and Perianal Conditions				
Perianal Fistula	No	208 (81.9)	202 (95.7)	<0.001
	Yes	46 (18.1)	9 (4.3)	
Hemorrhoid	No	213 (83.9)	192 (91.0)	0.022
	Yes	41 (16.1)	19 (9.0)	
Anal Fissure	No	249 (98.0)	208 (98.6)	0.652
	Yes	5 (2.0)	3 (1.4)	
Anal Abscess	No	242 (95.3)	208 (98.6)	0.045
	Yes	12 (4.7)	3 (1.4)	
Anal Stricture	No	252 (99.2)	210 (99.5)	0.674
	Yes	2 (0.8)	1 (0.5)	
Different Complications				
Fistula	No	149 (58.7)	196 (92.9)	<0.001
	Yes	105 (41.3)	15 (7.1)	
Strictures	No	187 (73.9)	208 (98.6)	<0.001
	Yes	66 (26.1)	3 (1.4)	
Adhesions	No	251 (98.8)	211 (100.0)	0.113
	Yes	3 (1.2)	0 (0.0)	
Perforation	No	244 (96.1)	209 (99.1)	0.043
	Yes	10 (3.9)	2 (0.9)	
Abscess	No	227 (89.4)	208 (98.6)	<0.001
	Yes	27 (10.6)	3 (1.4)	
Polyps	No	247 (97.2)	202 (95.7)	0.374
	Yes	7 (2.8)	9 (4.3)	
Bowel obstruction	No	238 (93.7)	210 (99.5)	0.001
	Yes	16 (6.3)	1 (0.5)	
Different Types of Surgeries				
Fistulotomy	No	243 (95.7)	210 (99.5)	0.009
	Yes	11 (4.3)	1 (0.5)	
Stricturoplasty	No	231 (90.9)	207 (98.1)	0.001
	Yes	23 (9.1)	4 (1.9)	
Abscess drainage	No	240 (94.5)	206 (97.6)	0.088
	Yes	14 (5.5)	5 (2.4)	
Ileocecal resection	No	253 (99.6)	209 (99.1)	0.457
	Yes	1 (0.4)	2 (0.9)	
Hemicolectomy	No	252 (99.2)	202 (95.7)	0.014
	Yes	2 (0.8)	9 (4.3)	
Proctocolectomy	No	248 (97.6)	211 (100.0)	0.025
	Yes	6 (2.4)	0 (0.0)	
Total colectomy	No	246 (96.9)	209 (99.1)	0.103
	Yes	8 (3.1)	2 (0.9)	
Subtotal colectomy	No	245 (96.5)	206 (97.6)	0.461
	Yes	9 (3.5)	5 (2.4)	
Small bowel resection	No	245 (96.5)	208 (98.6)	0.151
	Yes	9 (3.5)	3 (1.4)	
Ileocecectomy	No	246 (97.2)	209 (99.1)	0.157
	Yes	7 (2.8)	2 (0.9)	

**Table 6:** Difference between CD and UC patients considering the type of surgery.

		Diagnosis				
		CD		UC		*p*-Value
		Count	Column N %	Count	Column N %	
Fistulotomy	No	243	95.7%	210	99.5%	0.009
	Yes	11	4.3%	1	0.5%	
Stricturoplasty	No	231	90.9%	207	98.1%	0.001
	Yes	23	9.1%	4	1.9%	
Abscess drainage	No	240	94.5%	206	97.6%	0.088
	Yes	14	5.5%	5	2.4%	
Ileocecal resection	No	253	99.6%	209	99.1%	0.457
	Yes	1	0.4%	2	0.9%	
Hemicolectomy	No	252	99.2%	202	95.7%	0.014
	Yes	2	0.8%	9	4.3%	
Proctocolectomy	No	248	97.6%	211	100.0%	0.025
	Yes	6	2.4%	0	0.0%	
Total colectomy	No	246	96.9%	209	99.1%	0.103
	Yes	8	3.1%	2	0.9%	
Subtotal colectomy	No	245	96.5%	206	97.6%	0.461
	Yes	9	3.5%	5	2.4%	
Small bowl resection	No	245	96.5%	208	98.6%	0.151
	Yes	9	3.5%	3	1.4%	
Ileocecectomy	No	246	97.2%	209	99.1%	0.157
	Yes	7	2.8%	2	0.9%	

**Table 7:** Associations of different comorbidities with admission duration and surgical procedures.

	Duration of Longest Admission	Diabetes	Hyperlipidemia	Hypertension	Pancreatitis due to Therapy
Duration of Longest Admission	Not admitted	217 (55.1)	218 (53.3)	226 (53.9)	257 (55.7)
	1–3 days	30 (7.6)	30 (7.3)	33 (7.9)	34 (7.4)
	4–7 days	68 (17.3)	71 (17.4)	71 (16.9)	75 (16.3)
	8–14 days	47 (11.9)	50 (12.2)	50 (11.9)	53 (11.5)
	15 days–1 month	20 (5.1)	27 (6.6)	26 (6.2)	28 (6.1)
	>1 month	12 (3.0)	13 (3.2)	13 (3.1)	14 (3.0)
	*p*-value	0.316	0.146	0.352	0.066
Fistulotomy	Yes	25 (6.3)	24 (5.9)	25 (6.0)	26 (5.6)
	*p*-value	0.975	0.618	0.426	0.744
Stricturoplasty	Yes	18 (4.6)	18 (4.4)	19 (4.5)	19 (4.1)
	*p*-value	0.501	0.878	0.656	0.099
Abscess drainage	Yes	2 (0.5)	3 (0.7)	2 (0.5)	3 (0.7)
	*p*-value	0.463	0.354	0.140	0.678
Ileocecal resection	Yes	7 (1.8)	10 (2.4)	11 (2.6)	11 (2.4)
	*p*-value	0.673	0.520	0.172	0.871
Hemicolectomy	Yes	6 (1.5)	6 (1.5)	6 (1.4)	6 (1.3)
	*p*-value	<0.001	0.761	0.266	0.755
Proctocolectomy	Yes	6 (1.5)	6 (1.5)	6 (1.4)	6 (1.3)
	*p*-value	0.578	0.362	0.414	0.818
Total colectomy	Yes	8 (2.0)	10 (2.4)	9 (2.1)	10 (2.2)
	*p*-value	0.898	0.237	0.991	0.766
Subtotal colectomy	Yes	10 (2.5)	14 (3.4)	13 (3.1)	14 (3.0)
	*p*-value	<0.001	0.160	0.726	0.723
Small bowel resection	Yes	12 (3.0)	12 (2.9)	12 (2.9)	12 (2.6)
	*p*-value	0.330	0.194	0.245	0.744
Ileocecectomy	Yes	6 (1.5)	9 (2.2)	7 (1.7)	8 (1.7)
	*p*-value	0.289	0.262	0.212	0.001

## Data Availability

The original data presented in the study are openly available in FigShare at https://figshare.com/account/articles/30625409?file=59572733 (accessed on 7 November 2025).

## Abbreviations

The following abbreviations are used in this manuscript:

IBD	Inflammatory Bowel Disease
CD	Crohn’s Disease
UC	Ulcerative Colitis
BMI	Body Mass Index
NGHA	National Guard Health Affairs
PSC	Primary Sclerosing Cholangitis
